# Evaluation of stool-based testing to diagnose tuberculosis in children using the Truenat platform in routine settings in Nigeria

**DOI:** 10.1128/jcm.01041-25

**Published:** 2025-12-10

**Authors:** J. O. Olabamiji, K. Ochei, O. Daniel, R. Eneogu, A. Ihesie, D. Nongo, A. Agbaje, P. Dakum, E. Elom, A. Mwansasu, C. Colvin, N. Diaz, A. Adelekan, S. Oyelaran, C. Mensah, O. Odola, A. Olayemi, P. de Haas, E. Klinkenberg

**Affiliations:** 1 Institute of Human Virology Nigeria, International Research Centre of Excellence (IRCE), BAZE University 434008, Abuja, Nigeria; 2 USAID Nigeria, Abuja, Nigeria; 3 National Tuberculosis, Leprosy and Buruli Ulcer Control Programme, Abuja, Nigeria; 4 IDDS project, Washington, DC, USA; 5 Office of Infectious Disease, TB Division, Credence Management Solutions, LLC. Support contractor to USAID Bureau for Global Health 521559 , Washington, DC, USA; 6 Office of Sustainable Development, Health Division, Public Health Institute, Support contractor to USAID Bureau for Africa, Washington, DC, USA; 7 KNCV TB Foundation 26102, the Hague, the Netherlands; 8 Connect TB, the Netherlands, consultancy services provided for USAID TB team, Washington, DC, USA; The University of North Carolina at Chapel Hill School of Medicine, Chapel Hill, North Carolina, USA

**Keywords:** tuberculosis, Truenat, Xpert MTB/RIF Ultra, children, stool, hard-to-reach, childhood TB

## Abstract

The World Health Organization (WHO) recommends testing stool with Xpert MTB/RIF Ultra (Xpert-Ultra) as an initial diagnostic test for detection of tuberculosis (TB) and rifampicin resistance in children. The option of testing stool on the Truenat platform could benefit children and their caregivers in remote areas where GeneXpert is not available. We report the results of research to validate a protocol for processing and testing stool on Truenat using an adapted Simple One Step (SOS) stool method in routine settings in Nigeria. Stool specimens from children with presumptive TB were tested with Truenat MTB Plus (Truenat) and Xpert-Ultra using a comparative cross-sectional study design. A total of 510 children were enrolled and submitted a stool specimen. Of these, 482 (94.5%) had valid results on both platforms, 28 (5.8%) with MTB detected and 454 MTB not detected. Of the 28 with MTB detected, eight were detected on both platforms, seven on Truenat only, and 13 on Xpert-Ultra only. The concordance rate between Truenat and Xpert-Ultra was high at 95.8%. Significantly more non-determinate (error/invalid) results were observed with Truenat compared to Xpert-Ultra (4.7% versus 1.2% respectively; *P* < 0.001). Truenat can accurately detect MTB using 100 mg of stool with an adapted SOS stool protocol. Further optimizing extraction methods could reduce the frequency of non-determinate results. These results hold promise for expansion of childhood TB diagnosis services to hard-to-reach areas with limited infrastructure that are suitable for Truenat placement. Global scale-up of these alternative testing approaches will be necessary to close the gap in childhood TB case finding.

**IMPORTANCE:**

Following WHO recommendations, high TB burden countries have commenced testing stool on GeneXpert to improve TB case finding among children and others who may not easily produce sputum. In previous work, we adapted the Simple One Step (SOS) stool method for GeneXpert for the Truenat platform. In the present study, we validated the revised processing method under routine conditions in health care facilities in Nigeria. We tested stool from 510 children with presumptive TB on both the GeneXpert and Truenat platforms across a variety of health care settings. Concordance between Truenat and Xpert MTB detection was high at 95.8%, confirming stool can be tested on Truenat in routine services. Our study demonstrates that TB diagnostic testing for children can be provided in peripheral health facilities located in remote geographic areas with limited infrastructure where Truenat can be placed. Global scale-up will help further close the gap in childhood TB case finding.

## INTRODUCTION

The diagnosis of pulmonary tuberculosis (TB) in children is challenging because they often present with nonspecific signs and symptoms, and laboratory confirmation of their disease is difficult due to its paucibacillary nature and the challenge of obtaining sputum samples which require invasive procedures that must be performed by a trained provider, usually at a hospital. Since January 2020, the World Health Organization (WHO) recommends the use of Xpert MTB/RIF and Xpert MTB/RIF Ultra (Xpert) on stool as an initial diagnostic test for detection of TB and resistance to rifampicin (RIF) in children with signs and symptoms of pulmonary TB (1, 2). Since then, stool has been recognized as an alternative to sputum for TB diagnosis in children using the GeneXpert platform. This guidance also included recommendations for stool processing methods, including the Simple One Step (SOS) method now utilized in many settings where stool-based testing is available. This approach to childhood TB diagnosis was introduced in Nigeria in 2020 in selected states and scaled up nationwide in 2021. Between October 2020 and September 2023, a total of 50,774 children from 0 to 14 years old were tested using stool samples in 14 states where the approach was initially introduced, with *Mycobacterium tuberculosis* complex (MTB) detected in 2,440 (4.8%) children (3).

In the same guidelines that endorsed stool as a diagnostic test for children with presumptive pulmonary TB, WHO also recommended Nucleic Acid Amplification Tests (NAAT), collectively referred to as rapid molecular WHO-recommended diagnostics (mWRD), for TB diagnostic testing. These include the Truenat MTB, MTB Plus (Truenat), and MTB-RIF Dx assays (4) from Molbio Diagnostics (India) (5). These assays are used on sputum samples as initial diagnostic tests and can detect MTB within one hour, with RIF resistance determined by performing an additional one-hour test. Since 2023, Truenat has been introduced and scaled up for near point of care testing in multiple high burden countries, especially in remote areas where use of the Xpert platform may be limited due to infrastructure challenges and lack of reliable power supply (1). Existing studies demonstrate that routine use of the Truenat test on sputum in primary healthcare centers with limited infrastructure is feasible (6, 7).

Nigeria is an early adopter of Truenat for routine diagnosis of pulmonary TB (8, 9). An evaluation of initial implementation efforts concluded that deployment of Truenat to peripheral health facilities led to an increase in TB and DR-TB case detection and decreased turnaround time for diagnosis and TB treatment initiation (8). From November 2021 to September 2023, a total of 101,338 sputum samples were tested on Truenat, and 9,747 (9.6%) were MTB positive across 39 Truenat instruments in primary and secondary health facilities in 14 states. Given this successful implementation, the Nigeria National Tuberculosis, Leprosy, and Buruli Ulcer Control program (NTBLCP) decided to expand the use of Truenat throughout Nigeria. By June 2025, Nigeria had 372 Truenat and 513 GeneXpert instruments in use. Diagnostic sites use either GeneXpert or Truenat, depending on the available infrastructure. Choice of testing modality is defined by which test is available at the nearest diagnostic site to where an individual is first identified as having signs and symptoms of TB. In the context of this expansion, testing stool samples on the Truenat platform could increase access to bacteriological confirmation of TB in children in Nigeria and elsewhere.

The USAID-funded Infectious Disease and Detection Surveillance (IDDS) project collaborated with the Uganda Supranational Reference Laboratory (SNRL) and KNCV TB Foundation (KNCV) to adapt the SOS protocol, originally developed for preparation of stool for Xpert testing, for processing stool on the Truenat platform (10). To validate these procedures in a routine setting, IDDS worked with the USAID-funded TB Local Organization Network Region 3 (TB LON-3) project, implemented by the Institute of Human Virology (IHVN), the NTBLCP, and USAID/Nigeria to conduct a comparative cross-sectional study among children with presumptive TB.

## MATERIALS AND METHODS

### Study setting and population

A cross-sectional study was conducted in Oyo and Osun States, including 35 health centers with 34 linked laboratories (6 Truenat and 28 GeneXpert laboratories). Children aged 0–14 years identified as having presumptive TB at any of the study sites were eligible for participation. Caregivers were asked to provide consent for their child to participate; for children above 7 years of age, assent was also asked. Children with presumptive TB were identified and recruited from the directly observed therapy (DOT) clinic, out-patient department (OPD) clinic, malnutrition, child welfare, and immunization clinics. Children who were critically ill, in-patient, or living with HIV/AIDS (CLHA) who had a positive result on the Lipoarabinomannan assay (LAM) were excluded from the study due to ethical concerns about morbidity and mortality that could result from potential delays in treatment.

### Sample size and sampling strategy

The enrollment target was set to 500 children so that participating laboratories could gain operational experience with the adapted SOS stool protocol (10). We anticipated that at least 25 (5%) children among these would have an MTB positive result on Truenat and/or Xpert. The 5% is based on the average MTB positivity rate reported on a quarterly basis by the NTBLCP for children tested via stool-based testing in routine settings on GeneXpert (3).

A 5-stage sampling strategy was used to select the study sites. First, the Southwest Zone was purposively selected due to the presence of TB LON-3 project implementation sites. Of the four states in the Southwest Zone, three had Truenat instruments installed at diagnostic centers, and two (Oyo and Osun) were randomly selected. Third, all health facilities in these two states that notified at least five childhood TB cases between October 2022 to September 2023 were selected (*n* = 35). These 35 facilities represented all tiers of the healthcare system: 7 tertiary, 12 secondary, and 16 primary centers, including 1 private tertiary and 7 private secondary facilities. Fourth, the overall sample size of 500 was allocated to each facility proportionate to the size of their TB case notifications in children. At the final stage, 6 Truenat laboratories and 28 GeneXpert laboratories were selected to test the stool specimens based on their proximity to the 35 enrollment sites; each enrollment site was linked to a designated Xpert and Truenat laboratory. Of the 35 enrollment sites, 14 had a GeneXpert instrument installed and available to test samples, and none had a Truenat instrument.

### Training

The Nigerian study team members were first oriented on the SOS stool processing method adapted for Truenat by KNCV and the Uganda Supra National reference laboratory (SNRL) team who developed the protocol (10). Thereafter, they conducted training for healthcare workers, laboratory staff, and TB program coordinators from the study sites. The training team consisted of the principal investigator, a certified Truenat Superuser/Trainer, and study team members proficient in Xpert stool-based testing. The training curriculum included modules on identifying children with presumptive TB, sample collection, transportation, storage, and collection of quality data. Laboratory staff who were already familiar with the SOS stool processing method for Xpert and Truenat processing for sputum were trained specifically on the use of the SOS stool processing method adapted for Truenat.

### Specimen collection and transportation

**Fig 1:**
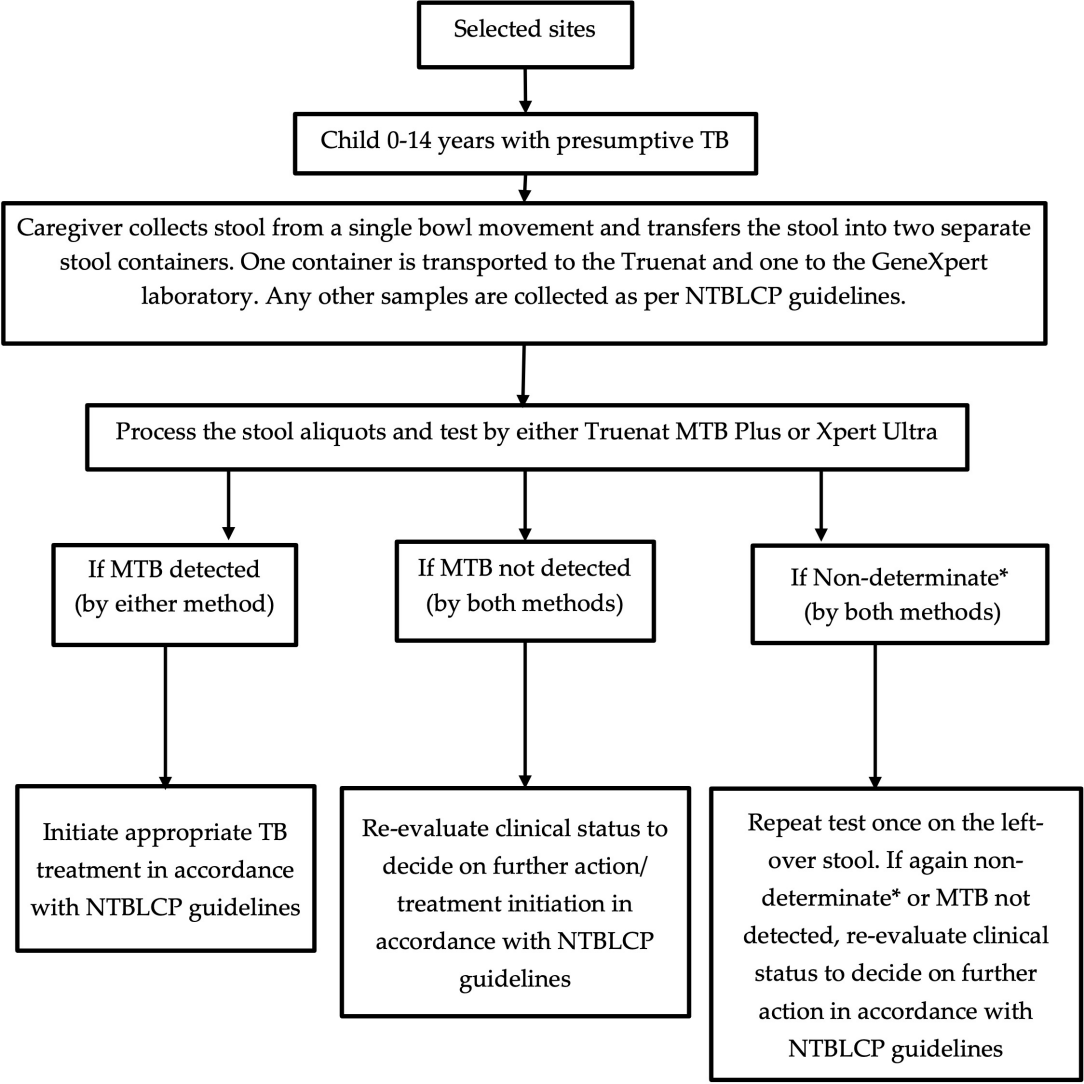
Flow diagram outlining study procedures. *non-determinate groups the results “invalid, error, or no result”. Abbreviations: TB, tuberculosis, Xpert, Xpert MTB/RIF-Ultra, MTB, *Mycobacterium tuberculosis complex,* NTBLCP, Nigeria National TB, Leprosy, and Buruli Ulcer Control program.

Basic study procedures are outlined in Fig. 1. Caregivers were given two stool collection containers with a small spoon and instructed to collect and submit stool from a single bowel movement into two separate containers. If a child was unable to produce stool on the spot, caregivers were asked to collect the stool samples at home and return them to the facility. The healthcare workers provided detailed instructions on stool collection at home, emphasizing the importance of returning the samples to the clinic as soon as possible, preferably on the day of collection (2). Trained healthcare workers collected other samples (e.g. sputum) as per NTBLCP guidelines.

All samples were transported in cold chain (4–8°C) using cooler boxes with icepacks to the designated laboratory as soon as possible but no later than 3 h after collection (as per routine practice) by a dedicated specimen transporter trained by the study team. One container was transported to the designated GeneXpert and one to the designated Truenat laboratory. There was no specific order for assigning the two specimens to either the Truenat or Xpert laboratory.

### Specimen processing and testing

Upon receipt at the laboratory, stool samples were warmed to room temperature before processing (2, 11). The consistency of the stool specimen was classified using the seven categories of the Bristol Stool Chart (1–5 formed/semi-formed, 6–7 liquid).

Approximately 800 mg of formed/semi-formed stool or 2 mL of liquid stool was processed and tested using the Xpert MTB/RIF Ultra (Xpert-Ultra) cartridge according to the SOS stool Xpert processing method (12) at Xpert testing sites.

Samples sent to Truenat laboratories were processed per the adapted SOS stool Truenat processing protocol (10). For formed/semi-formed stool, 100 mg was transferred into the 2.5 mL Lysis buffer (LB) bottle. For liquid stool, 0.5 mL of the stool was transferred into the LB bottle without removing any buffer. After adding the stool to the LB, the bottle was shaken vigorously for 30 s, then left to stand at room temperature for 5 min. Thereafter, 2 mL of the clear supernatant was transferred into the Trueprep Auto Cartridge for further preparation on the Trueprep Auto sample preparation system. The DNA elute was removed from the cartridge and tested on the Truenat MTBplus chip using the TrueLab instrument, and subsequent testing was done on Truenat RIF chip if MTB was detected, following NTBLCP routine SOP for sputum testing (13).

Both Xpert and Truenat use semi-quantitative outcomes which are linked to a range of cycle threshold (Ct) values. The following outcomes are provided for both the Xpert-Ultra and Truenat assay: MTB not detected, MTB detected high, MTB detected medium, MTB detected low, and MTB detected very low. Xpert-Ultra has one additional category (MTB trace detected). Both Truenat and Xpert-Ultra include the categories of RIF detected, not detected, or indeterminate for RIF testing outcomes. Both tests have three categories of non-determinate results: error, invalid result, or no result. In all instances of non-determinate results, repeat testing was conducted on both Xpert-Ultra and Truenat using leftover stool specimens, if available. After testing, the remaining specimens were stored at −80°C for up to 6 months to allow for additional testing as needed. In case of retesting, stools were thawed to room temperature.

The study team processed all respiratory samples, sputum or nasogastric aspiration (NGA) using the routine NTBLCP SOPs. These samples were processed on either the Xpert-Ultra or Truenat assay depending on what was available at the nearest laboratory (14).

During the data analysis phase (October 2024), stool samples that initially tested MTB positive on Xpert-Ultra (800 mg) but were MTB not detected on Truenat (100 mg) were retested using 100 mg on both Truenat and Xpert-Ultra, if sufficient leftover stool was available. This was done to assess if observed discordance was linked to the amount of stool used (800 versus 100 mg).

### Differential diagnosis

TB diagnosis was confirmed bacteriologically or clinically by the facility clinician based on Truenat and Xpert-Ultra test results and the NTBLCP diagnostic and treatment algorithm for children (15).

### Data capture and analysis

Demographic and clinical characteristics were recorded for each participant, as well as sample collection details, results of all laboratory tests performed, and the eventual TB diagnosis. Data capture was done at each site using a dedicated data management system in KoboCollect that was designed specifically for the study (KoboToolbox, open source). Weekly reports were compiled by the study team to track enrollment of children, sample collection and receipt, and laboratory results. On-site supervision visits were conducted by IHVN to address any implementation challenges through on-the-job mentoring and validation of the data received from the study sites. Analysis was conducted in Stata (StataCorp LLC, version 15). Descriptive analysis was done for characteristics of study participants. Concordance between test results using the Xpert-Ultra and Truenat assays was assessed using cross-tabulations and percentage of test results in agreement. Where relevant, χ square testing was used to test whether observed differences were significant. The main objective was to compare the concordance of MTB detection between the Truenat MTB Plus and Xpert-Ultra assays. In addition, we compared the detection of RIF resistance between the two assays.

## RESULTS

### Characteristics of children included

Between 12 February and 21 March 2024, a total of 510 children with presumptive TB were enrolled in the study and provided stool samples. Table 1 presents the characteristics of the children. The male-to-female ratio was 1:1, and just over half (56.7%) of the children were under 5 years old. Slightly more children were enrolled in Osun state compared with Oyo state, 286 versus 224 children, respectively. Among the 510 children who submitted stool samples, 34 children also provided a respiratory sample (31 spontaneous sputum and 3 NGAs). A chest X-ray was done for 38 children.

**TABLE 1:** Characteristics of children included in the study^*a*^

	n	%
Gender		
Female	243	47.6%
Male	267	52.4%
Age group		
0–4 years	289	56.7%
5–9 years	134	26.3%
10–14 years	87	17.1%
State		
Osun	286	56.1%
Oyo	224	43.9%
Diagnostic tools		
Stool	510	100.0%
Sputum/NGA	34	6.7%
CXR taken	38	7.5%

^*a*^ n, number; NGA, nasogastric aspiration; CXR, chest X-ray.

### Laboratory test results

#### Xpert-Ultra testing

**Fig 2:**
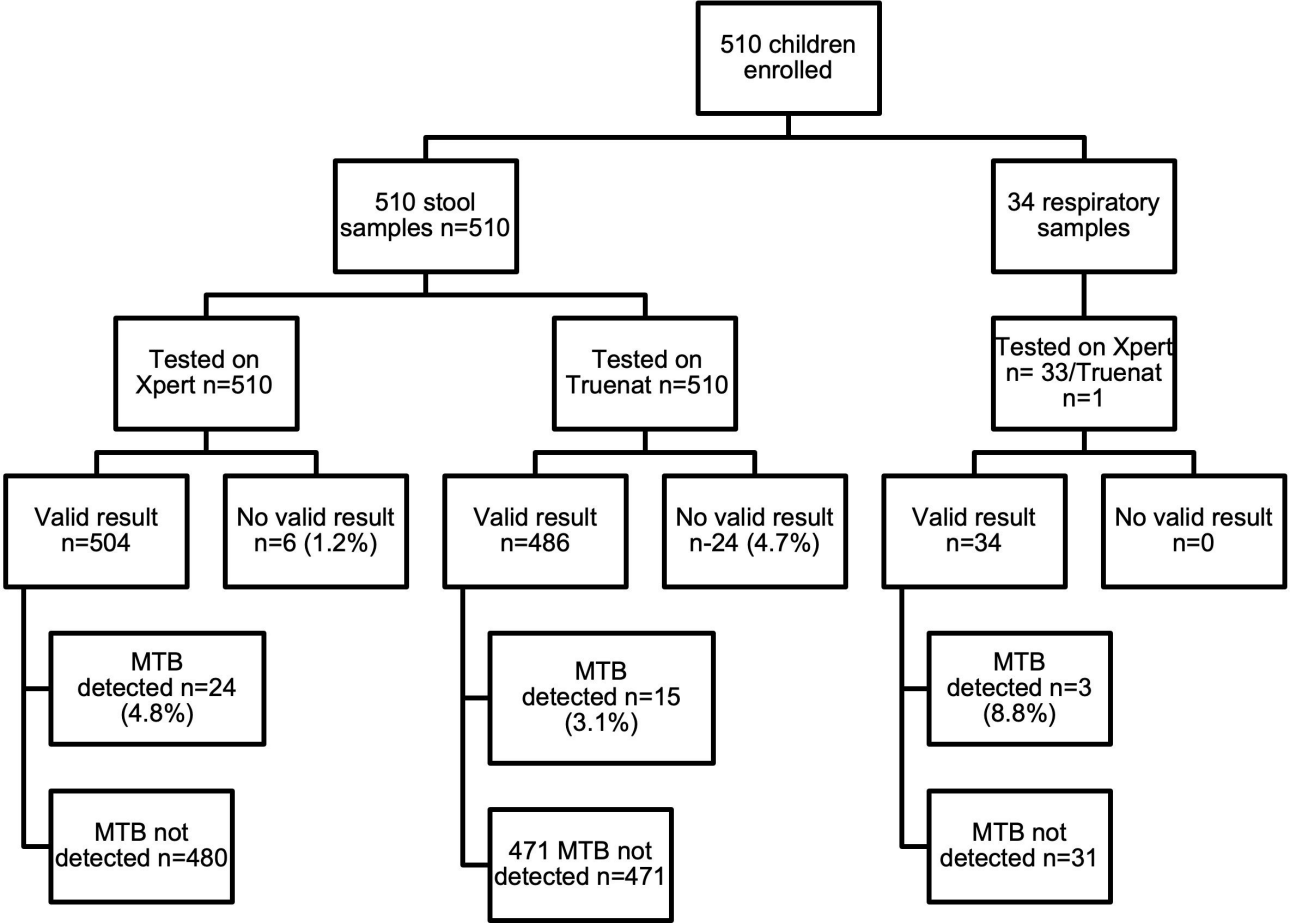
Flow diagram of type of samples collected, and the final test result of both Treunat and Xpert (after repeat testing). MTB, *Mycobacterium tuberculosis* complex*; n*, number; Xpert, Xpert MTB/RIF-Ultra; Truenat is Truenat MTB Plus.

All 510 stool samples were tested using the Xpert-Ultra assay (Fig. 2). Of these initial tests, 493 (96.7%) yielded valid results (469 MTB not detected, 24 MTB detected), while 17 had non-determinate results (3 error code 5007, 1 error code 5006, 2 no result, and 11 invalid). A repeat Xpert-Ultra test was conducted for 14 out of the 17 non-determinate results, resulting in 11 MTB not detected and 3 invalid results. No repeat test was done for the remaining three samples due to insufficient leftover stool. Combining the initial and repeat Xpert-Ultra test results (Table 2), 480 (94.1%) were MTB not detected, 24 (4.7%) had MTB detected (7 MTB low, 8 MTB very low, and 9 MTB trace), and 6 (1.2%) were non-determinate results (4 invalid, 2 with no result). Among the 24 samples with MTB detected, 15 showed no mutation of resistance to RIF, whereas nine resulted in RIF indeterminate, all of which were MTB trace detected, which by default is RIF indeterminate.

**TABLE 2:** Summary of final testing results on stool for Xpert-Ultra and Truenat MTB plus among 510 children enrolled in the study^*a*^

		MTB result					RIF result		
Diagnostic test	Tests conducted	MTB not detected	MTB detected	MTB yield^*c*^	Non-determinate result (error/invalid/ No Result)	% non-determinate results^*b*^	RIF indeterminate result	% RIF indeterminate results	Remark on RIF indeterminate results
Truenat- MTB Plus (100 mg)	510	471	15	2.9%	24	4.7%	8	53.3%	All RIF indeterminate are MTB detected very low
Xpert-Ultra (800 mg)	510	480	24	4.8%	6	1.2%	0/9^*d*^	0%/37.5^*d*^%	All RIF indeterminate are MTB trace detected^*d*^

^*a*^ MTB, *Mycobacterium tuberculosis *complex; RIF, rifampicin; Xpert, Xpert MTB/RIF-Ultra; mg, milligram.

^*b*^ Difference significant at *P *< 0.05 (Chi-square statistic 11.1273, *P *= 0.000851).

^*c*^ Difference not significant.

^*d*^ For Xpert trace RIF resistance cannot be detected due to the test composition, therefore they always result in RIF indeterminate. Therefore, we provided two figures, including and excluding the RIF indeterminate because of trace. For Xpert Ultra, if trace are excluded, no RIF indeterminate would have been detected in Xpert.

#### Truenat testing

All 510 stool samples were tested on the Truenat platform with the MTB Plus assay (Fig. 2). Of these initial tests, 477 (93.5%) had a valid Truenat result (462 MTB not detected, 15 MTB detected), while 33 had non-determinate results (two errors [codes 2 and 3] and 31 had invalid results). A repeat test was conducted for 30 out of the 33 stools with non-determinate results, resulting in 9 MTB not detected and 21 non-determinate results (1 error, 1 no result, and 19 invalid). No repeat test was done for the remaining three (one error, two invalid) due to insufficient leftover specimen. Combining the initial and repeat Truenat results (Table 2), 471 (92.4%) were MTB not detected, 15 (2.9%) were MTB detected (1 MTB medium, 1 MTB low, 13 MTB very low), and 24 (4.7%) were non-determinate (two error, 21 invalid, one no result).

Among the 15 samples with MTB detected by Truenat, seven showed no mutation for resistance to RIF, while eight resulted in RIF indeterminate, all of which were MTB detected very low.

#### Respiratory samples

Valid test results were obtained for all 34 respiratory samples (Fig. 2), with 33 tested on Xpert-Ultra and 1 tested on Truenat. One Xpert-Ultra test was initially returned as invalid, but upon repeat testing, resulted in MTB not detected. Of the 34 valid results, 3 (8.8%) were MTB detected (2 MTB very low and 1 MTB high); none showed mutation associated with RIF resistance.

#### Overall MTB detection: stool and respiratory samples

Combining the results of all stool and respiratory samples, MTB was detected in 32 (6.3%) children. From the three children confirmed to have MTB by respiratory samples, two were also MTB detected on stool (one on both the Truenat and Xpert-Ultra assay and one just on Xpert-Ultra, all MTB detected very low). One child was MTB detected in the respiratory sample only. Another child had MTB detected on stool but not on the respiratory sample; for this child, stool Xpert-Ultra was MTB detected very low, while Truenat was MTB not detected. The other 28 children confirmed MTB positive had MTB detected only by stool, as no respiratory sample was available.

### Concordance of Truenat and Xpert-Ultra test results

Table 3 shows the concordance between Truenat and Xpert-Ultra results on stool. Of the 482 stool specimens with a valid result on both assays, 462 (95.9%) were concordant, of which 454 (98.3%) tested MTB negative, and 8 had MTB detected on both tests. Combining results for both assays, MTB was detected in a total of 31 stool specimens; 13 had MTB detected only on Xpert-Ultra and 7 only on Truenat, while for eight, MTB was detected by both assays. Additionally, three specimens had MTB detected by Xpert-Ultra but did not have valid test results on Truenat. Using MTB detection on Xpert-Ultra as the established standard for comparison, the negative percent agreement was 98.5% (454/461), and the positive percent agreement was 38.5% (8/21).

**TABLE 3:** Concordance between semi-quantitative results of Xpert-Ultra using 800 mg of stool and Truenat MTB plus using 100 mg of stool^*a*^

	Semi-quantitative results from Xpert-Ultra							
Semi-quantitative results from Truenat MTB Plus	MTB Not detected	MTB detected Medium	MTB detected Low	MTB detected Very Low	MTB Trace detected	Invalid	No Result	Total
MTB Not detected	454	0	3	3	7	3	1	471
MTB detected Medium	1	0	0	0	0	0	0	1
MTB detected Low	0	0	0	1	0	0	0	1
MTB detected Very Low	6	0	4	2	1	0	0	12
Error^*b*^	1	0	0	1	0	0	0	2
Invalid	18	0	0	1	1	1	1	22
Total	480	0	7	8	9	4	2	510

^*a*^ MTB, *Mycobacterium tuberculosis *complex; Xpert-Ultra, Xpert MTB/RIF-Ultra; Truenat is Truenat MTB PLus.

^*b*^ Error code E003, E002.

Comparison of non-determinate results between Truenat and Xpert-Ultra revealed that, after repeat testing, six (1.2%) stools were non-determinate on Xpert-Ultra, while 24 (4.7%) stools were non-determinate on Truenat (Table 2). This difference was statistically significant (*P* < 0.001).

### Retesting of discordant results between Xpert-Ultra and Truenat

Out of the 13 stools with Xpert-Ultra MTB detected using 800 mg of stool that were MTB not detected on Truenat using 100 mg of stool, five could be retested on both Xpert-Ultra and Truenat using 100 mg of the leftover specimen. For the remaining eight specimens, there was not sufficient stool for retesting. Table 4 describes the five retesting results. Two out of the five Xpert-Ultra MTB detected on 800 mg also had MTB detected on 100 mg of stool (one MTB very low, one MTB trace). These same two specimens resulted in MTB detected on Truenat 100 mg (MTB detected low) upon retesting. For the remaining three stools, upon retesting, MTB was not detected either on Truenat or Xpert-Ultra using 100 mg of stool, despite being Xpert-Ultra MTB detected (MTB low, MTB trace) on the initial 800 mg stool sample.

**TABLE 4:** Semi-quantitative re-testing results for Xpert-Ultra and Truenat for those stools initially tested MTB positive on Xpert-Ultra (800 mg), but MTB not detected on Truenat (100 mg)^*a*^^,^^*b*^

Child no	Sex	Age group (years)	Initial Xpert-Ultra MTB result (800 mg)	Re-test Xpert-Ultra MTB result (100 mg)	Initial Truenat MTB result (100 mg)	Re-test Truenat MTB result (100 mg)
Child 1	Male	0c4	**MTB detected trace**	MTB not detected	MTB not detected	MTB not detected
Child 2	Male	0–4	**MTB detected low**	MTB not detected	MTB not detected	MTB not detected
Child 3	Female	10–14	**MTB detected very low**	**MTB detected very low**	MTB not detected	**MTB detected very low**
Child 4	Female	5–9	**MTB detected low**	**MTB Trace detected**	MTB not detected	**MTB detected very low**
Child 5	Female	0–4	**MTB detected trace**	MTB not detected	MTB not detected	MTB not detected

^*a*^ no, number; mg, milligram; MTB, *Mycobacterium tuberculosis* complex; Xpert-Ultra, Xpert MTB/RIF-Ultra; Truenat is Truenat MTB PLus.

^*b*^ Boldface indicates result that are concordant among the different tests done.

## DISCUSSION

Our study shows that routine testing of stool on the Truenat platform is feasible across a range of service delivery settings, and the Truenat assay can be used to detect MTB in stool samples from children using the adapted SOS stool method developed by the Uganda SNRL and KNCV (10). Concordance between Truenat and Xpert-Ultra results on stool was high at 95.8%. We observed significantly more non-determinate test results with Truenat compared to Xpert-Ultra; the proportion of non-determinate results for MTB detection after one repeat was 4.7%, which is below the threshold of 5% set by WHO for this quality control indicator (1) However, for the detection of RIF resistance, the indeterminate rate was as high as 53.3%.

Though the overall concordance was high (98.5%), relatively low concordance was observed for Truenat against Xpert-Ultra for MTB positive stool at 38.5%. In 13 children, MTB was confirmed only by Xpert-Ultra, while in seven children, it was confirmed only by Truenat. All stools that showed a discordant result for MTB were detected as low bacilli load samples. This result is not surprising, as children are known to have paucibacillary disease compared to adults. For paucibacillary samples, there is a possibility that the stool portion taken for testing contained bacteria levels below the limit of detection, resulting in MTB not detected, whereas another portion might have had a bacteria concentration slightly above the limit of detection, with a positive MTB test result. This phenomenon, which we call the “lucky draw” effect, has also been observed in sputum samples (16). This effect is relevant to the recent WHO recommendation for concurrent testing of stool and respiratory samples for children to avoid missing TB in this vulnerable paucibacillary population (1). Additionally, recently released WHO guidance for TB prevalence surveys in community settings recommends two Xpert-Ultra tests to increase the chance of finding TB among study participants likely to have paucibacillary or subclinical TB (17). Incremental yield when testing two stools was also shown by Tiemersma and colleagues (18) and was highest among samples with a low bacillary load. Since the limit of detection for Truenat and Xpert-Ultra is similar, our discordant test results may be due to the different stool portions tested by the different assays.

Earlier studies have shown that testing stool on Xpert-Ultra results in a slightly higher rate of non-determinate results compared to sputum (19, 20). This is likely due to the nature of stool, including the presence of debris that can more easily clog the cartridge and cause an error or invalid result (2, 12). Additionally, stool may contain a higher concentration of inhibitors, which may lead to non-determinate results (19, 21–23).

In this study, we observed a significantly higher rate of non-determinate results for MTB on Truenat compared with Xpert-Ultra, which affected observed concordance. For three participants, MTB was detected in stool by Xpert-Ultra, but no valid final Truenat result was obtained. A higher non-determinate rate was also observed when testing clinical samples in phase 2 of the study in Uganda (10). This difference may be attributed to the smaller volume of lysis buffer for Truenat (2.5 mL) compared with the Xpert assay (8 mL), which makes it more difficult to transfer a clean supernatant into the Truenat cartridge due to limited maneuvering space for pipetting. While the volume of stool used for Truenat is smaller (100 mg) compared with the Xpert assay (800 mg), the increased volume of lysis buffer in Xpert not only provides more maneuvering space but may also dilute any inhibitors present in the sample. Further research is needed to investigate whether using a larger volume of Truenat lysis buffer could reduce the number of non-determinate results. Additionally, the impact of differences in chemical composition of the lysis and sample reagent buffers, which play a role in the first processing step, needs further investigation.

In addition to the lysis buffer, Truenat test kits include a liquefaction buffer. According to the protocol of the manufacturer, a total of four drops of liquefaction buffer should be added during sputum processing (24). The liquefaction buffer contains tris (2-carboxyethyl) phosphine (TCEP), which reduces disulfide bonds in RNA and DNA extraction procedures, helping prevent oxidation of nucleic acids and maintaining their integrity during isolation. Anecdotal evidence from this study suggests that adding four drops of liquefaction buffer to the stool-lysis buffer mixture can help reduce the number of non-determinate results. At St. Peters General Hospital in Oyo State, one of the larger Truenat testing sites, the study team encountered cartridge clog errors in four samples at the DNA extraction stage. These stool samples contained traces of mucus. During repeat testing, four drops of liquefaction buffer were added as recommended for mucoid sputum, resulting in valid results for all four stool samples. Continuing this practice for mucoid stool led to no further non-determinate results. Though we could not assess this systematically during the study, we believe adding four drops of liquefaction buffer could reduce the non-determinate rate. One benefit of this approach is that procedures for stool and sputum processing on Truenat would be better aligned, which in turn would simplify staff training.

Moreover, we note that the amount of stool tested differed between the two assays. We used approximately 800 mg of stool for the Xpert-Ultra test and around 100 mg for Truenat. Given the paucibacillary nature of samples from children, the 8-times larger stool volume used for Xpert-Ultra testing may have increased the chance of detecting MTB. To explore this hypothesis, we retested samples that were MTB detected on Xpert-Ultra (800 mg) but MTB not detected on Truenat (100 mg) using 100 mg aliquots for both Xpert-Ultra and Truenat. Results showed that two out of five stools that were initially MTB negative on Truenat returned an MTB detected result upon retesting. This illustrates the above-described “lucky draw” effect that can be observed among samples with low bacillary load. However, in previous work, the experiments conducted by KNCV and SNRL/Uganda to determine the optimal stool volume for Truenat processing, no direct relationship between the stool volume and MTB positivity rate was observed, and concordance between Xpert-Ultra on 100 and 600 mg of stool was high at 98.1% (10). Furthermore, a study on the robustness of the SOS stool processing method did not suggest that a smaller volume of stool would significantly lower MTB positivity rate (21).

In addition to the higher non-determinate rate of results for MTB in Truenat, we observed a higher indeterminate rate for RIF resistance testing in Truenat. This high RIF indeterminate rate is also observed for sputum Truenat testing (25). The same issue was also reported with the former Xpert MTB/RIF cartridge in some low-prevalence settings. However, in the Xpert-Ultra cartridge, the manufacturer improved the detection of RIF resistance by using a melting temperature technology. To our knowledge, for Truenat, this technology is not used. Furthermore, Truenat uses a different set of probes to determine RIF resistance. These differences might explain the observed high indeterminate rate for RIF resistance in our study. In their latest update to the diagnostic guidance, WHO advised that resistance is inferred rather than detected for Truenat MTB-RIF Dx (1).

Nearly 60% of the children enrolled in this routine setting were under 5 years of age. This age group has the largest case detection gap and is most likely to benefit from the availability of stool testing at the point of care (26). Stool-based TB testing is increasingly a game-changer for expanding access to a bacteriological confirmatory TB test for young children. Data from routine implementation of stool-based Xpert testing from Zambia (27), Nigeria (3), and Malawi (28) indicate that a larger proportion of children under 5 years with presumptive TB were able to provide a stool sample for bacteriological testing compared with the proportion for whom sputum was available, often after invasive and unpleasant procedures. Zambia and Nigeria also reported a significant increase in annual childhood TB notifications following the introduction of stool-based testing. Notably, stool-based testing was introduced as part of a comprehensive package of measures to enhance TB case detection among children, including strengthening capacity for clinical diagnosis among providers. Adults who have difficulty producing sputum may also benefit from stool testing in addition to children (19, 27, 29, 30).

We excluded critically ill children and those with HIV and a positive LAM test result, which may have biased the study population toward less severe disease. The exclusion of these children was deemed necessary to avoid asking parents to sign consent forms in a stressful situation and to avoid delays in differential diagnosis and treatment initiation. Since our objective was to determine the concordance between Xpert-Ultra and Truenat on stool samples, and not the actual TB burden, we are less concerned about this potential bias.

Truenat is being rolled out for routine use in high-burden countries, especially in remote settings where the use of the GeneXpert platform is not always feasible due to infrastructure challenges. The Truenat instrument has an internal battery, which can protect testing capacity when facing short power failure or unstable power supply. In addition, the Truenat instrument is less sensitive to dust, which is an important advantage in more remote environments. In countries like Nigeria, where most healthcare facilities regularly experience power outages and are in hard-to-reach areas with limited infrastructure, Truenat enhances access to TB diagnosis services. Data from Nigeria showed that the deployment of Truenat to test sputum led to an increase in TB and DR-TB case detection in peripheral facilities, decreased turnaround time, and improved time to TB treatment initiation (8). Having the option to test stool samples on Truenat would further increase access to TB diagnosis, especially for younger children, as shown in our study results. We conducted a modeling exercise to evaluate the health impact, health-system costs, and cost-effectiveness of using Truenat on stool samples compared to the standard of care for pediatric TB diagnosis in primary care settings in Nigeria. From this modeling, we concluded that implementing stool-based Truenat testing has the potential to increase access and reduce direct health system costs associated with childhood TB diagnosis in routine health care settings in Nigeria (31).

Our findings confirm that stool samples can be processed on the Truenat platform using an adapted SOS method with high concordance with Xpert-Ultra testing of stool. Further optimizing extraction methods could reduce the frequency of non-determinate results. Being able to test stool also on Truenat provides hope for improving childhood TB diagnostic services in peripheral health facilities, particularly in hard-to-reach areas with limited access to infrastructure. Global scale-up of this and other innovative case detection approaches is required to close the gap in childhood TB case finding. More studies are needed to build the evidence base for WHO to include stool-based testing on Truenat as a part of their recommendations for TB diagnosis.
